# Intergenerational Pathogen-Induced Diapause in Caenorhabditis elegans Is Modulated by *mir-243*

**DOI:** 10.1128/mBio.01950-20

**Published:** 2020-09-22

**Authors:** Carolaing Gabaldón, Marcela Legüe, M. Fernanda Palominos, Lidia Verdugo, Florence Gutzwiller, Andrea Calixto

**Affiliations:** a Centro Interdisciplinario de Neurociencia de Valparaíso, Valparaíso, Chile; b Programa de Doctorado Genómica Integrativa, Universidad Mayor, Santiago de Chile, Chile; c Programa de Doctorado en Ciencias, Mención Neurociencia, Universidad de Valparaíso, Valparaíso, Chile; University of Texas Health Science Center at Houston

**Keywords:** *Caenorhabditis elegans*, DualRNAseq, defense, intergenerational, miRNA, pathogen, small RNAs

## Abstract

The interaction and communication between bacteria and their hosts modulate many aspects of animal physiology and behavior. Dauer entry as a response to chronic exposure to pathogenic bacteria in Caenorhabditis elegans is an example of a dramatic survival response. This response is dependent on the RNA interference (RNAi) machinery, suggesting the involvement of small RNAs (sRNAs) as effectors. Interestingly, dauer formation occurs after two generations of interaction with two unrelated moderately pathogenic bacteria. Therefore, we sought to discover the identity of C. elegans RNAs involved in pathogen-induced diapause. Using transcriptomics and differential expression analysis of coding and long and small noncoding RNAs, we found that *mir-243-3p* (the mature form of *mir-243*) is the only transcript continuously upregulated in animals exposed to both Pseudomonas aeruginosa and Salmonella enterica for two generations. Phenotypic analysis of mutants showed that *mir-243* is required for dauer formation under pathogenesis but not under starvation. Moreover, DAF-16, a master regulator of defensive responses in the animal and required for dauer formation was found to be necessary for *mir-243* expression. This work highlights the role of a small noncoding RNA in the intergenerational defensive response against pathogenic bacteria and interkingdom communication.

## Abstract (precis)

Persistent infection of the bacterivore nematode C. elegans with bacteria such as P. aeruginosa and S. enterica makes the worm diapause or hibernate. By doing this, the worm closes its mouth, avoiding infection. This response takes two generations to be implemented. In this work, we looked for genes expressed upon infection that could mediate the worm diapause triggered by pathogens. We identify *mir-243-3p* as the only transcript commonly upregulated when animals feed on P. aeruginosa and S. enterica for two consecutive generations. Moreover, we demonstrate that *mir-243-3p* is required for pathogen-induced dauer formation, a new function that has not been previously described for this microRNA (miRNA). We also find that the transcriptional activators DAF-16, PQM-1, and CRH-2 are necessary for the expression of *mir-243* under pathogenesis. Here we establish a relationship between a small RNA and a developmental change that ensures the survival of a percentage of the progeny.

## INTRODUCTION

Caenorhabditis elegans has a close evolutionary relationship with bacteria (1), as it has naturally evolved exposed to microbes from the soil that can either be their food source or be a threat (2–4). In laboratory settings, C. elegans has been fed for decades on the standardized bacteria Escherichia coli OP50, and only in recent times has our understanding of this nematode-bacterium relationship evolved from a simple static organism/substrate pair to a dynamic model in which the host’s and microbe’s performance changes throughout their association (5). Moreover, their ability to recognize and defend themselves from potential pathogens has likely been shaped by its continuous encounters with different types of bacteria, and thus when confronted with infectious microbes, C. elegans can avoid them by displaying complex behavioral, endocrine, and immune responses (6, 7). The worm response is triggered by specific molecules secreted by bacteria such as toxic pigments from Pseudomonas aeruginosa PA14 (8), cyanide from P. aeruginosa PAO1 (9), and serrawettin W2, produced by Serratia marcescens (10). The behavioral response is mediated by ASJ and ASI neurons through the activation of the DAF-7/TGF-β (transforming growth factor β) pathway (7) in concert with neuropeptidergic control of innate immunity (11). When avoidance is not possible and worms are exposed to highly pathogenic bacteria, they die within 24 h (12). In contrast, when confronted with mildly pathogenic bacteria for two or more generations, a percentage of the population enters diapause, forming the dauer larvae, an alternative stress-resistant larval stage that does not feed and thus is able to properly avoid pathogen infection (13). As pathogen-induced dauer formation (PIDF) depends on the RNA interference (RNAi) machinery (13), we propose that for its initiation, PIDF requires the expression of a specific set of small RNAs (sRNAs), and in the long-term, the maintenance of sRNA expression across more than one developmental cycle. This accumulation of sRNAs could generate molecular footprints that will predispose the upcoming generations of worms to enter diapause, thus ensuring the survival of a percentage of the total population. In this way, the sustained but dynamic communication between host and pathogen enables the worm’s development and reproduction over consecutive generations (13).

The molecules signaling diapause entry in the second generation after pathogen exposure are unknown. This work focuses on the discovery of sRNAs involved in the response of C. elegans to chronic pathogenic infection that leads to defensive dauer formation. Here we show that *mir-243-3p* (the mature form of *mir-243*) is overexpressed in animals exposed to two unrelated pathogens and is needed to mount intergenerational pathogen-induced diapause formation. We also show that transcription factors DAF-16, PQM-1, and CRH-2 are required for the expression of the mature form of *mir-243.* Furthermore, PQM-1 and CRH-2 are also needed for dauer formation under pathogenesis. This work reveals an intergenerational role for *mir-243* in the defense against pathogens and highlights the importance of small RNAs as mediators of long-term survival strategies.

## RESULTS

### Global gene expression changes in intergenerational chronic exposure to pathogens.

**FIG 1:**
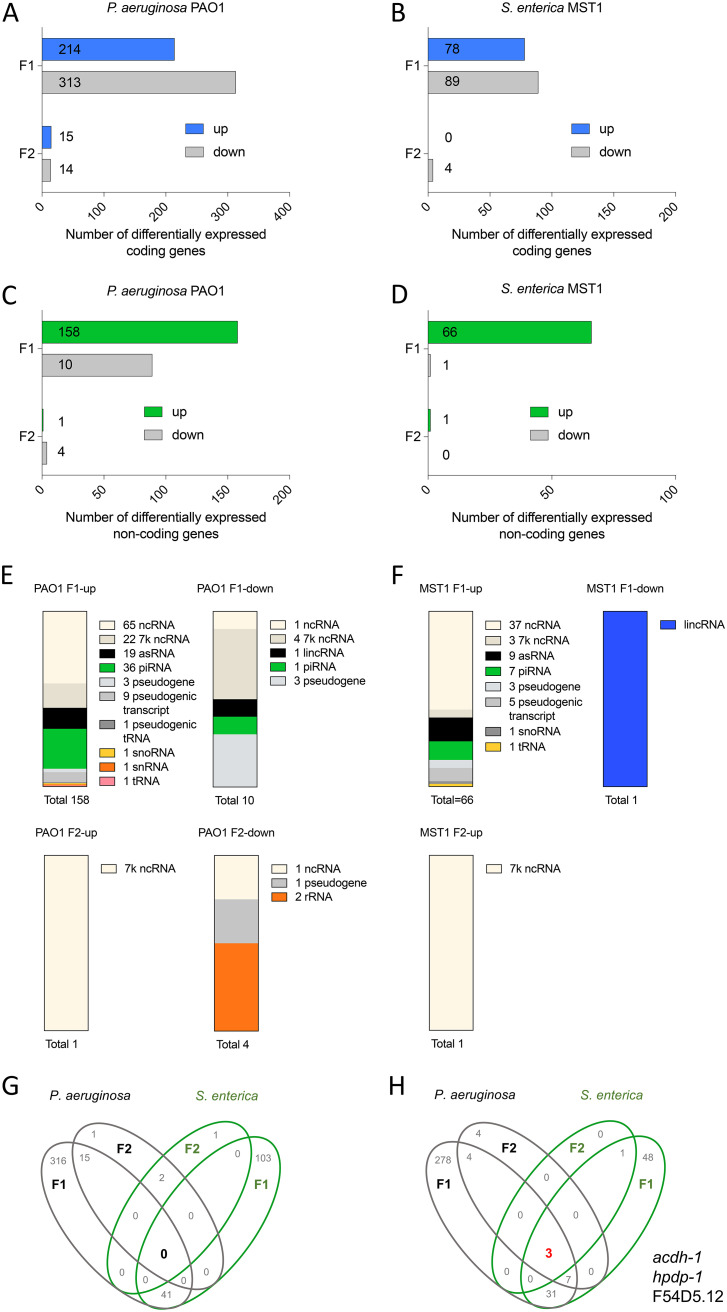
Global analysis of differential mRNA gene expression of an intergenerational infection paradigm. (A to D) Number of poly(A) RNA coding genes (A and B) and noncoding genes (C and D) differentially expressed on P. aeruginosa PAO1 (A and C) and S. enterica serovar Typhimurium MST1 (B and D) in two generations. (E and F) Type and abundance of noncoding RNAs on P. aeruginosa PAO1 and S. enterica MST1 in two generations. (G and H) Venn diagram representation of shared and unique genes upregulated (G) and downregulated (H) in each generation and on each pathogen.

Developmental and behavioral plasticity likely emerges from broad, complex gene expression changes at different molecular levels. To reveal RNA profiles underlying the intergenerational diapause entry (13), we performed a transcriptomic analysis of two generations of synchronized C. elegans in the larval stage 2 (L2). Animals were grown on the two diapause-inducing bacteria, P. aeruginosa PAO1 and Salmonella enterica serovar Typhimurium MST1, and on E. coli OP50, which does not trigger dauer formation. We aimed to find transcriptomic changes elicited by both pathogens in the two generations that could explain PIDF. Sequencing was performed on both mRNA and small RNA libraries generated by separate methods (see Materials and Methods). In this first result, we will address poly(A)^+^ RNAs while sRNAs will be addressed in the next section. To detect mRNAs and other polyadenylated transcripts, mRNA libraries were poly(A) selected [poly(A)^+^]. We performed differential gene expression analysis of animals feeding on pathogenic bacteria, using nonpathogenic E. coli OP50 as a reference. We considered differentially expressed (DE) those genes with a log_2_ fold change of >1, adjusted *P* value (padj) <0.05 by DeSeq, and *P* < 0.05 by EdgeR (see Data Set S1 and Tables S1 and S2 in the supplemental material). Differentially expressed poly(A)^+^ RNAs included coding and noncoding transcripts (Fig. 1A to D). Among noncoding transcripts, we found Piwi-interacting RNAs (piRNAs), 7k noncoding RNA (ncRNA), pseudogenes, tRNAs, long intergenic noncoding RNAs (lincRNAs), antisense RNAs (asRNAs), small nucleolar RNA (snoRNA), small nuclear RNA (snRNA), and ribosomal RNA (rRNA) (Fig. 1E and F). Surprisingly, transcriptional changes in coding and noncoding poly(A)^+^ RNAs were much larger in abundance and diversity in the first generation (F1) than in the second generation (F2) of animals fed with either pathogen (Fig. 1A to F).

10.1128/mBio.01950-20.2 TABLE S1 mRNA coding and noncoding genes differentially expressed under pathogenic conditions. Upregulated genes expressed in F1 and F2 of animals feeding on P. aeruginosa PAO1 or S. enterica MST1. Download Table S1, XLSX file, 0.02 MB. Copyright © 2020 Gabaldon et al. 2020 Gabaldon et al. This content is distributed under the terms of the Creative Commons Attribution 4.0 International license.

10.1128/mBio.01950-20.3 TABLE S2 mRNA coding and noncoding genes differentially expressed in pathogenic conditions. Downregulated genes expressed in F1 and F2 of animals feeding on P. aeruginosa PAO1 or S. enterica MST1. Download Table S2, XLSX file, 0.01 MB. Copyright © 2020 Gabaldon et al. 2020 Gabaldon et al. This content is distributed under the terms of the Creative Commons Attribution 4.0 International license.

10.1128/mBio.01950-20.9 DATA SET S1 Differential expression analysis of expressed mRNA genes in two generations of animals exposed to P. aeruginosa PAO1 and S. enterica MST1 by DeSeq and EdgeR. Download Data Set S1, XLSX file, 0.7 MB. Copyright © 2020 Gabaldon et al. 2020 Gabaldon et al. This content is distributed under the terms of the Creative Commons Attribution 4.0 International license.

Poly(A)^+^ RNAs were overexpressed or repressed in a generation- and/or pathogen-specific manner (Table S3) and did not share upregulated coding or noncoding poly(A)^+^ sequences that were common to both pathogens and both generations (Fig. 1G). Notwithstanding, we found coincidences for the repression of three genes: *acdh-1*, *hphd-1*, and F54D5.12 (Fig. 1H), in both pathogens and across the two generations. All coding genes differentially expressed in the F2 were also up- or downregulated in the F1, with no new genes of this kind turned on or off selectively in the F2 (Table S3).

10.1128/mBio.01950-20.4 TABLE S3 Pathogen-specific and shared differentially expressed genes under each condition. Download Table S3, XLSX file, 0.02 MB. Copyright © 2020 Gabaldon et al. 2020 Gabaldon et al. This content is distributed under the terms of the Creative Commons Attribution 4.0 International license.

**FIG 2:**
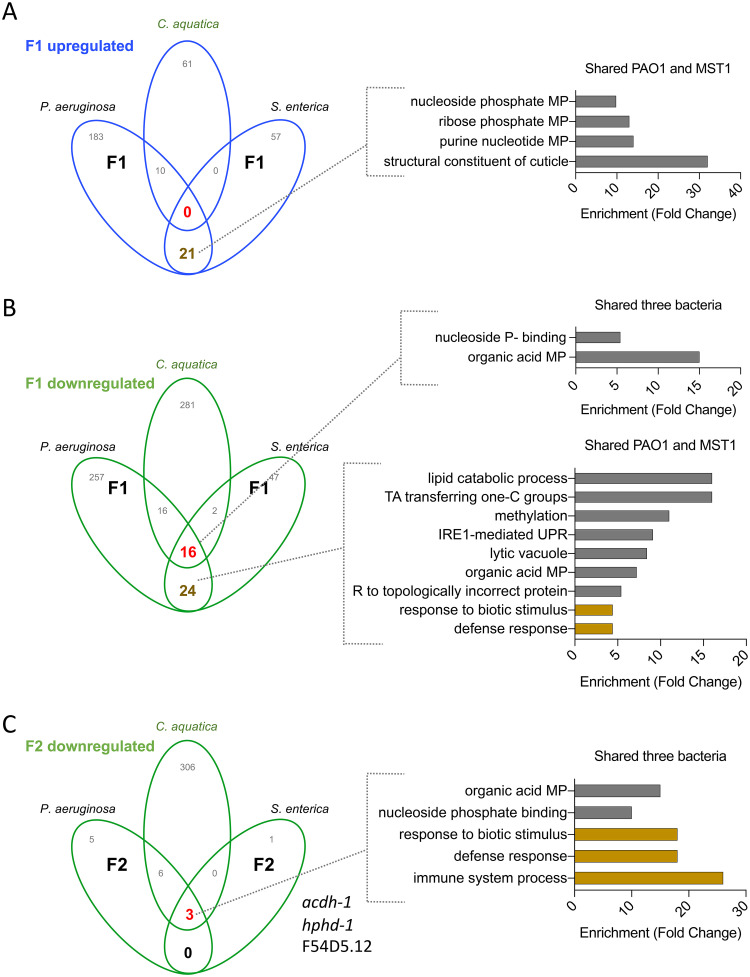
Coincidences in upregulated (A) and downregulated (B and C) genes of animals feeding on pathogens and those reported for *C. aquatica* (14) for one (A and B) and two (C) generations after change from E. coli OP50. Each figure includes GO enrichments for genes shared with *C. aquatica* and those shared between pathogens. MP, metabolic process; P, phosphate; TA, transferase activity; C, carbon; UPR, unfolded protein response; R, response.

To distinguish between changes caused by food switch from E. coli to other bacteria from changes induced by long-lasting pathogenic exposure, we compared the expression profiles of animals feeding on P. aeruginosa and S. enterica in the F1, to those reported for Comamonas aquatica, a nourishing food for C. elegans (14). Upregulated coding genes were only present between pathogens (“pathogen-exclusive” in Fig. 2A), but repressed genes were found in worms exposed to the three bacteria (Fig. 2B). These 16 downregulated genes were all enriched in Gene Ontology (GO) terms related to metabolic processes. This reveals a common “food switch factor” caused by changing diet from E. coli to other food sources despite their pathogenic potential. We then compared the expression changes produced in the second generation (F2) of worms exposed to P. aeruginosa and S. enterica to those produced by worm’s first encounter with C. aquatica (14). Surprisingly, *acdh-1*, *hphd-1*, and F54D5.12 (Fig. 1H) remained downregulated in both pathogens and *C. aquatica* (Fig. 2C). Therefore, when animals are switched from E. coli to pathogens, a mixed transcriptional response involving regulation of metabolic and immune response is triggered. However, in the long term, the response is specified and reduced to a small subset of downregulated genes that are common between pathogens and nutritious food (see Fig. S1C in the supplemental material), showing that the transcriptional response prior to dauer formation in the F2 reflects a defensive response to pathogenic conditions intertwined with an ongoing metabolic transformation.

10.1128/mBio.01950-20.1 FIG S1 Enrichment by GO term of upregulated (A and B) and downregulated (D and E) in animals feeding on P. aeruginosa PAO1 and S. enterica serovar Typhimurium MST1 in two generations. (C and F) Summary of shared GO terms in F1 and F2 in upregulated (C) and downregulated (F) genes. MP, metabolic process; TA, transferase activity; HA, hydrolase activity; N, nitrogen; C, carbon; R, response; P, phosphate; UPR, unfolded protein response. Download FIG S1, PDF file, 0.2 MB. Copyright © 2020 Gabaldon et al. 2020 Gabaldon et al. This content is distributed under the terms of the Creative Commons Attribution 4.0 International license.

In addition, to relate transcriptional changes exclusively induced by pathogen exposure to processes of physiological relevance for PIDF, we examined enriched GO terms in up- and downregulated genes, in each generation, and in each bacterium (Fig. S1A and B and S1D and E). Upregulated genes in the F1 on both pathogens shared enrichment in structural components of the cuticle (Fig. S1A to C). S. enterica could be tested only in the F1 because there was only one differentially expressed gene in the F2. In P. aeruginosa, both generations displayed GO enrichment, with the F2 specifically enriched in genes involved in defense against biotic stress (Fig. S1A and C). The latter was significantly and specifically enriched in animals fed on P. aeruginosa for two generations (Fig. S1D to F). Taken together, these results further prompt the idea that in the long-term, C. elegans can adapt to the pathogenic encounter, overcoming the general response to diet change and keeping specific biological changes that may aid their survival, such as cuticle, metabolism, defense, and dauer reprogramming.

Global analysis of mRNA overexpression shows that changes are dissimilar for animals feeding on S. enterica serovar Typhimurium MST1 and P. aeruginosa PAO1 throughout the two generations. Since worms can enter diapause feeding on both P. aeruginosa and S. enterica, the decision that gives rise to PIDF is not solely reliant on changes in mRNA expression but also on other levels of regulation. To further understand its underlying molecular causes, we analyzed changes in the sRNA repertoire in both bacteria and generations.

### sRNA expression in two generations of C. elegans exposed to bacterial pathogens.

**FIG 3:**
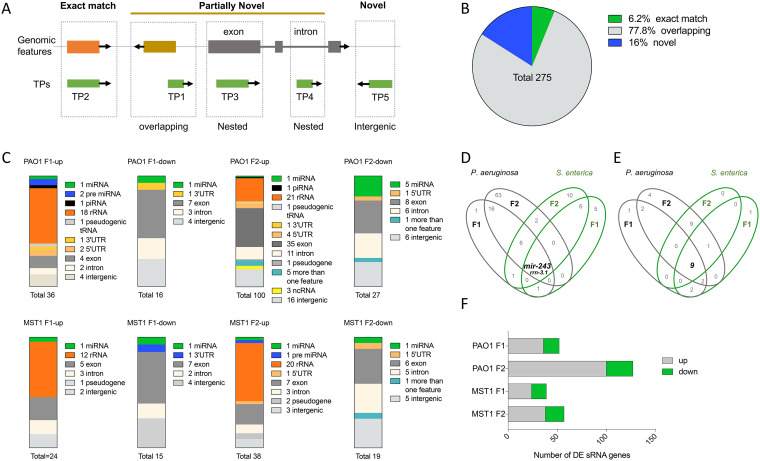
Global analysis of differential small RNA gene expression of an intergenerational infection paradigm. (A) Representation of transcriptional unit designation. (B) Genomic context of sRNA genes differentially expressed under pathogenic conditions. (C) Type and abundance of sRNA genes differentially expressed on P. aeruginosa PAO1 and S. enterica MST1 in two generations. (D and E) Venn diagram representation of shared and unique genes overexpressed (D) and repressed (E) in each generation and on each pathogen. (F) Number of sRNA genes differentially expressed on P. aeruginosa PAO1 and S. enterica MST1 in two generations.

Small RNAs (sRNAs) are broad regulators of gene expression (15–17) and key candidates to modulate inter- and transgenerational environmental adaptation (13, 18, 19). We aimed to unbiasedly identify known and novel sRNAs expressed when animals are fed on each bacterium through two consecutive generations. To do that, we defined candidate sRNA loci based on transcriptional peak (TP) coordinates (defined in Materials and Methods). These TPs were used for the downstream transcriptome sequencing (RNA-seq) analysis. Then we compared those genomic loci with annotated features to classify them as known (matching an annotated sRNA), novel (located in intergenic regions), or partially novel (unannotated but overlapping or nested within a feature, see Fig. 3A and Data Set S2). Considering all differentially expressed genes in either pathogen, 6.2% were known features, 77.8% were partially novel sequences, and 16% were novel TPs (Fig. 3B). Known differentially expressed TPs in pathogen-fed worms include pre-miRNAs and mature microRNAs (miRNAs). Partially novel TPs were nested in intronic or exonic segments of coding genes or overlapping with 5′ and 3′ untranslated region (UTR) ends. The TPs nested in noncoding transcripts were found in rRNAs and 21-ur RNAs. We also found pseudogenic transcripts, tRNAs, and novel TPs within intergenic regions (Fig. 3C and Tables S4 and S5). Interestingly, from all expressed TPs, *mir-243*-*3p* along with another 11 TPs nested within the *rrn-3.1* ribosomal gene were upregulated in both generations of worms fed on P. aeruginosa PAO1 and S. enterica MST1 (Fig. 3D and Table S6). In contrast, the pre*mir-70* and another nine novel TPs were downregulated similarly in both bacteria and generations (Fig. 3E and Table S6, shared all conditions). Despite these, most TPs were specifically DE in response to strain MST1 or PAO1 or to both pathogens but only in one generation. For example, *mir-51* was exclusively upregulated in the F1 of animals feeding on PAO1 (Table S6, generation-specific), but *21ur-6043* was upregulated in both generations on PAO1 (Table S6, bacterium-specific). Interestingly, four miRNAs (*mir-1*, *mir-48*, *mir-256*, and *mir-257*) were downregulated only in the second generation on P. aeruginosa PAO1 (Table S4). One of these miRNAs, *mir-48*, a *let-7* family (*let-7*Fam) member is known to be repressed on the more virulent P. aeruginosa strain PA14 (20). These results show that sRNA expression in the context of long-term pathogen exposure is mostly specific to one generation or one pathogen. However, *mir-243-3p* is overexpressed across conditions, suggesting that it may function as a common effector in PIDF. As the response to PAO1 in the F2 was the largest in terms of the numbers of TPs both up-, and downregulated (Fig. 3F), the following experiments were performed using P. aeruginosa PAO1.

10.1128/mBio.01950-20.5 TABLE S4 Small RNAs differentially expressed in pathogenic conditions. Up- and downregulated sRNAs in the F1 and F2 of animals feeding on P. aeruginosa PAO1. Download Table S4, XLSX file, 0.02 MB. Copyright © 2020 Gabaldon et al. 2020 Gabaldon et al. This content is distributed under the terms of the Creative Commons Attribution 4.0 International license.

10.1128/mBio.01950-20.6 TABLE S5 Small RNAs differentially expressed in pathogenic conditions. Up- and downregulated sRNAs in the F1 and F2 of animals feeding on S. enterica MST1. Download Table S5, XLSX file, 0.01 MB. Copyright © 2020 Gabaldon et al. 2020 Gabaldon et al. This content is distributed under the terms of the Creative Commons Attribution 4.0 International license.

10.1128/mBio.01950-20.7 TABLE S6 Pathogen-specific and shared differentially expressed genes under each condition. Download Table S6, XLSX file, 0.01 MB. Copyright © 2020 Gabaldon et al. 2020 Gabaldon et al. This content is distributed under the terms of the Creative Commons Attribution 4.0 International license.

10.1128/mBio.01950-20.10 DATA SET S2 Differential expression analysis and genomic context of expressed sRNA genes in two generations of animals exposed to P. aeruginosa PAO1 and S. enterica MST1. Download Data Set S2, XLSX file, 0.2 MB. Copyright © 2020 Gabaldon et al. 2020 Gabaldon et al. This content is distributed under the terms of the Creative Commons Attribution 4.0 International license.

### *mir-243* is necessary for diapause formation under pathogenesis.

**FIG 4:**
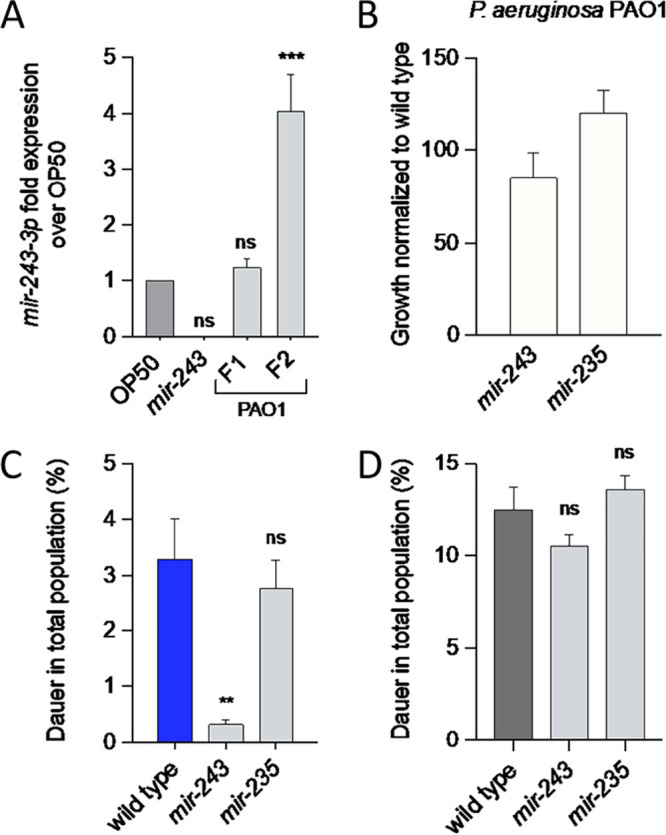
Expression of mature *mir-243* and role in pathogen-induced diapause. (A) Quantification of *mir-243-3p* expression in animals feeding on P. aeruginosa PAO1 for two generations. (B to D) Growth (B), dauer formation on pathogens (C), and dauer formation by starvation (D) of *mir-243* mutant animals. ns, nonsignificant; **, *P* < 0.0021; ***, *P* < 0.0002.

To test the requirement of *mir-*243 on PIDF, we first quantified by reverse transcription-PCR (RT-PCR) the relative amounts of mature *mir-243-3p* in each generation of animals feeding on P. aeruginosa PAO1 compared to those feeding on E. coli OP50. RNA was extracted from L2 worms fed on P. aeruginosa and E. coli in the F1 and F2, as was done for the transcriptomic analysis. We found that *mir-243-3p* appeared upregulated by fourfold in the second generation of worms feeding on P. aeruginosa PAO1 and onefold change in the F1, and as expected, *mir-243* (*n4759*) mutants were unable to express mature *mir-243* (Fig. 4A). To further explore the role of *mir-243* in PIDF, we tested whether mutant animals for *mir-243* (*n4759*) were able to form dauers on P. aeruginosa PAO1 in the second generation. We also tested as reference animals with a deletion in *mir-235* (*n4504*), a microRNA involved in nutritional-related L1 diapause (21), which was not DE under pathogenesis. *mir-243* and *mir-235* mutants were fed on P. aeruginosa PAO1 for two generations. The population growth and appearance of dauers were quantified in the F2 and compared to wild-type animals. Growth on P. aeruginosa PAO1 was not affected by either mutation (Fig. 4B), but only *mir-243* mutants were found to form significantly less dauers than wild-type or *mir-235* animals when fed on P. aeruginosa PAO1 (Fig. 4C). Therefore, these mutations do not increase susceptibility to pathogens, as revealed by growth, but since none of them were deficient in dauer formation under starvation (Fig. 4D), this result importantly suggests that *mir-243* has a specific role in dauer formation under pathogenesis.

We have shown that *mir-243* is required for PIDF, and thus, we explored its potential molecular targets. Interestingly, *mir-243* is known to activate the exo-RNAi pathway by binding RDE-1, triggering the production of secondary siRNAs. The Y47H10A.5 mRNA has been revealed to be repressed by this mechanism (20). Even though *mir-243 i*s upregulated in all PIDF conditions, Y47H10A.5 is not differentially expressed in our data. Therefore, we tested the hypothesis that some downregulated mRNA under pathogenesis could be *mir-243* targets. Using the IntaRNA tool version 2.0 (22), we computed the expected RNA-RNA interactions among *mir-243* and our downregulated mRNAs. We found that *mir-243* has the potential for binding 26 of our 136 downregulated genes with high complementarity (seed ≥12) and strong negative free energy (NFE). Even though Y47H10A.5, a validated *mir-243-3p* target, is not differentially expressed in our data, we computed the interaction with the same parameters and found that Y47H10A.5 has higher NFE and shorter seed that our candidate targets (Table S7).

10.1128/mBio.01950-20.8 TABLE S7 Putative targets of *mir-243* among genes downregulated in pathogenic conditions. Download Table S7, XLSX file, 0.01 MB. Copyright © 2020 Gabaldon et al. 2020 Gabaldon et al. This content is distributed under the terms of the Creative Commons Attribution 4.0 International license.

### DAF-16 and other transcriptional activators regulate the expression of *mir-243* under pathogenesis.

**FIG 5:**
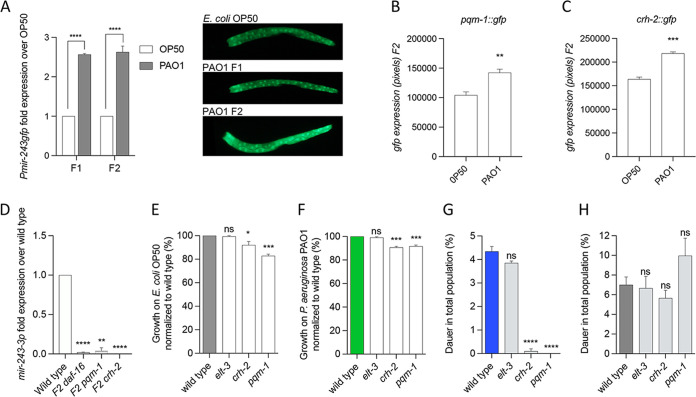
Transcriptional factors required for *mir-243* expression and pathogen-induced diapause formation. (A) Quantification of *mir-243* promoter expression by GFP in animals feeding on P. aeruginosa PAO1 compared to E. coli OP50 and representative photos. (B and C) Quantification of expression of PQM-1 (B) and CRH-2 (C) by GFP expression on animals fed on pathogens. (D) Quantification by RT-PCR of *mir-243-3p* expression in animals fed wild-type and *daf-16*, *pqm-1*, and *crh-2* mutant animals. (E to G) Growth on E. coli OP50 (E) and P. aeruginosa PAO1 (F) and dauer formation (G) in the second generation of animals. (H) Dauer formation on starvation of the wild type and mutants of transcription factors. *, *P* < 0.03; **, *P* < 0.0021; ***, *P* < 0.0002; ****, *P* < 0.0001; ns, nonsignificant.

To study whether the increase of mature *mir-243* in animals feeding on pathogens in the F2 could be a result of transcriptional activation, we quantified the fluorescence of a strain expressing *gfp* under the *mir-243* promoter (VT1474). We measured *gfp* expression in L2 worms grown on pathogens for two generations and compared it with those grown on E. coli OP50. Animals feeding on pathogens overexpress *Pmir-243*::*gfp* compared to E. coli OP50 controls in both generations (Fig. 5A), suggesting that the exposure to pathogens activates the transcription of *mir-243* in concordance with our previous expression results (Fig. 4A). However, the mechanism behind *mir-243* transcriptional activation is unknown. A number of transcription factors (TFs) promote the expression of their targets under stress and infection (23–25). It has been previously reported that *mir-243* and members of the *let-7*Fam are the miRNAs with the highest number of interactions to TFs in the C. elegans genome (26). We specifically tested the roles of three transcriptional regulators, DAF-16, PQM-1, and CRH-2 in *mir-243-3p* expression. The reasons for choosing these three regulators are explained below. In our paradigm, DAF-16/FOXO transcriptional activator localizes into the nuclei of animals exposed to P. aeruginosa PAO1 prior to diapause formation (13). PQM-1 resides in the nucleus regulating the expression of DAF-16-associated elements (DAE), avoiding dauer formation (27). Finally, we included CRH-2 because it is a direct target negatively regulated by the *let-7*Fam of microRNAs (28), which have been involved in the response to pathogenesis by the P. aeruginosa PA14 strain (20). In our experiments, *mir-48*, a *let-7*Fam miRNA, was downregulated in PAO1 in the F2 (Table S4). Under this logic, *crh-2* could be indirectly upregulated through *mir-48* downregulation. To give further ground to this selection, we tested whether *pqm-1* and *crh-2* promoter expression was higher in pathogens compared to nonpathogenic conditions. Using strains expressing *gfp* under promoters for *pqm-1* or *crh-2,* we observed that the expression of both TFs is upregulated in animals fed with P. aeruginosa PAO1 for two generations compared to those fed on E. coli OP50 (Fig. 5B and C), as we have previously reported for DAF-16 (13). To study whether these transcriptional regulators are necessary for the expression of *mir-243* under pathogenesis, we extracted RNA from *daf-16* (*m27*), *pqm-1* (*ok485*), and *crh-2* (*gk3293*) mutants in the F2 of worms fed with P. aeruginosa PAO1 and quantified the expression of *mir-243-3p* over wild-type animals. All three mutants fed on P. aeruginosa for two generations completely lacked *mir-243-3p* expression (Fig. 5D). These results show that DAF-16, PQM-1, and CRH-2 transcription factors are needed for the expression of *mir-243-3p* in the second generation of animals exposed to pathogens.

Because *mir-243* loss affects the ability of animals to enter diapause under pathogenesis, we further explore whether *crh-2* and *pqm-1* mutants also failed to form dauers under infection but formed normal amounts of dauers under starvation. Additionally, we tested whether these mutants were able to grow on E. coli and P. aeruginosa to wild-type extents. As a control, we used a mutant of *elt-3*, a transcriptional activator with broad expression in the animal*. daf-16* mutation causes animals to be unable to form dauers (29) and could not be tested. All TF mutants grew well on E. coli OP50 and to a similar extent as wild-type animals on pathogenic bacteria (Fig. 5E and F). Interestingly, *crh-2* and *pqm-1* mutants were unable to enter diapause under pathogenesis after two generations (Fig. 5G) but formed normal amounts of dauers under starvation (Fig. 5H), while *elt-3* animals formed normal amounts under both pathogenesis and starvation (Fig. 5G and H). Taken all together, these results suggest that the role of *crh-2* and *pqm-1* TFs is specific to PIDF and that an expression signaling cascade, including CRH-2, PQM-1, and DAF-16 upstream of *mir-243* expression is triggered by the long-term interaction of worms with the mild pathogen P. aeruginosa PAO1.

## DISCUSSION

Survival strategies to cope with environmental challenges rely on the genetic plasticity of organisms. In this work, we dissected the transcriptomic differences and similarities between worms feeding on three different bacteria. Two of these bacteria, P. aeruginosa PAO1 and S. enterica serovar Typhimurium MST1, elicit dauer entry as a defense strategy in the second generation (13). Differential gene expression analysis allowed us to identify *mir-243-3p* (the mature form of *mir-243*) as the only common upregulated sRNA in animals fed on these pathogens for two generations. Moreover, *mir-243* mutant animals do not perform PIDF despite not being dauer defective under starvation. Finally, we examined the roles of transcription factors DAF-16, PQM-1, and CRH-2 on *mir-243-3p* expression under pathogenesis. All three were shown to be required for *mir-243* expression. Furthermore, in contrast to dauer-defective DAF-16, the CRH-2 and PQM-1 transcription factors are specifically required for PIDF but not for dauer formation under starvation.

### Most transcriptional changes are unique to the encounter with each pathogen.

Gene expression is highly variable and dependent on environmental and physiological factors. C. elegans transcriptional profile is modified when animals are exposed to a new bacterial diet. The bacterial diets can be nutritious food such as Comamonas aquatica (14), nonpathogenic such as Bacillus subtilis (30), or pathogenic such as Shigella flexneri (31), P. aeruginosa PA14 and Staphylococcus aureus (32, 33). Temperature and diet changes trigger gene expression changes associated with defensive responses and metabolism (30). Environmental conditions can change the phenotypes of subsequent generations (34–36), suggesting that transcriptomic modifications can be inherited in the progeny. Accordingly, we speculate that the greater abundance in poly(A)^+^ transcripts on the F1 may reflect the response to a novel food source (being their first time not feeding on E. coli OP50; Fig. 2) and generalized stress and immune responses to pathogenesis. Therefore, the second generation of worms forced to feed on pathogens may have adapted to the pathogenic bacteria through inherited signals, narrowing the transcriptional changes to a more reduced transcriptional response (Fig. S1) (37).

In this work, we discovered transcriptional changes that could explain the defensive decision to enter the dauer program as a response to pathogenesis (13). We notice that two different bacterial pathogens trigger the same phenotypic response in C. elegans but display few underlying transcriptomic similarities. We think that this could be an indicator of high functional specificity and result from a finely tuned long-term communication between the bacteria and host. Clustering of genes by function (38) revealed large differences between animals fed on the two bacteria. In the wild, C. elegans is mostly found in the dauer stage, a strategy used to maximize the animal’s survival by ensuring their dispersal to new food sources (39). Therefore, dauer entry may be a convergent phenotypic outcome driven by a plethora of stimuli and transcriptional regulatory pathways that, under adverse circumstances, allows the survival of the species.

### Small RNAs and pathogenesis.

Several works have explored the role of sRNA in infection (40, 41). Among them, microRNAs have been reported to be involved in the innate immune response of C. elegans against infection with bacterial pathogens, and even with eukaryotes such as the opportunist yeast Candida albicans (42). For example, *let-7* regulates the innate immune response by targeting intestinal SDZ-24 when fed on the highly pathogenic P. aeruginosa PA14 (43). Moreover, other candidate targets of *let-7* and *let-7*Fam of microRNAs may include components of the PMK-1/p38 innate immune pathway (20). Likewise, *mir-67* mutants exhibited reduced pathogen avoidance behavior, apparently due to a dysregulation in *sax-7* targeting (44, 45). Others like *mir-70* and *mir-251/mir-252* mutants possess enhanced survival to P. aeruginosa PA14 infections, indicating that these miRNAs negatively regulate the immune response (46). Supporting this idea, we found that *mir-70* was systematically downregulated in animals feeding on both P. aeruginosa and S. enterica.

We describe here that a single sRNA, *mir-243*, was the only upregulated transcriptional coincidence among animals fed on pathogens for two generations and, as a result, was required for pathogen-induced dauer formation. As mentioned, *mir-243* has an unusual association with RDE-1, an Argonaut protein known to be a siRNA acceptor (47), suggesting that *mir-243* may induce mRNA destabilization depending on the RNAi machinery. In accordance with that, we have previously shown that RDE-1, along with other RNAi effectors, are needed for the induction of dauer formation under pathogenesis (13). Predicted targets of *mir-243* within downregulated genes were almost perfectly complementary, suggesting that the mechanism by which *mir-243* induces silencing in the context of PIDF could be also related to siRNA pathways as previously reported for other targets (47). Our approach allows us to narrow the spectra of possible *mir-243* targets in our experimental paradigm. *mir-243* can potentially target many genes for silencing. Côrrea et al. (47) reported 1,835 upregulated genes in the *mir-243* mutant (microarray analysis comparing adult wild-type and mutant worms, fold change ≥ 2, *P* < 0.05). We found 24 common genes between published upregulated genes in *mir-243* mutant (47) and downregulated genes in our data sets. Three of the genes (C14B1.3, *acs-2*, and *mrp-2*) have high negative free energy (NFE) of interaction and high sequence complementarity (greater than or equal to 12 bp), becoming good candidates for future validation studies. The biological validation of predicted *mir-243* targets, as well as the role of downregulated microRNAs such as *mir-70* in PIDF remains to be elucidated. Notwithstanding, our findings support the hypothesis that the response of C. elegans to different pathogens is accompanied by dynamic changes in the activity of miRNAs. In this work, we found that *mir-243* is involved in the response of C. elegans to infection with both pathogens, P. aeruginosa PAO1 and S. enterica MST1. Moreover, as the same miRNA is triggering similar effects on different pathogens, this may imply that changes in a single microRNA can induce similar phenotypic outputs, like dauer formation, but distinct molecular cascades to achieve it.

### Transcription factors involved in diapause formation as a defensive strategy.

A number of transcription factors activate the transcription of their targets under stress and infection. DAF-16 has been reported to regulate the expression of genes involved in defense when exposed to pathogenic bacteria such as P. aeruginosa PA14 (48), S. enterica strain 1344, Yersinia pestis strain KIM5, and S. aureus MSSA476 (49). In the pathogen-induced dauer formation paradigm, DAF-16 localizes in the nuclei of animals exposed to P. aeruginosa PAO1 prior to diapause formation (13). In this work, we show that *mir-243* expression requires intact DAF-16. Furthermore, both PQM-1, which affects the expression of DAF-16 in the nucleus (27), and CRH-2, which has been proposed to be regulated by *let-7* miRNA (28), are needed for *mir-243* expression under pathogenesis, and their loss impairs PIDF. Although we did not carry out an exhaustive analysis of TFs related to the formation of dauer by pathogens, an extensive list of TFs that interact with *mir-243* and that could regulate it directly or indirectly is available in the work of Martinez et al. (26). In this work, we did not find differentially expressed genes whose mutations have been described before as conducing to abnormal dauer formation (*Daf*). This suggests at least two things, that the expression of *Daf* genes does not necessarily change at the level of mRNAs or that the dauer program under pathogenesis is molecularly different from starvation-induced diapause (an abiotic stressor).

Dauer formation upon pathogenesis is likely a multistep process that involves the sensing, initiation, and establishment of the pathogenic state. The transcriptional difference in poly(A)^+^ genes of animals feeding on either pathogen during the F1 is much larger than in the F2 compared to their usual E. coli OP50 food. We speculate that the F1 response is pleiotropic and involves (i) the first encounter with new bacteria and diet and (ii) the response to a pathogen. Importantly, the worm expression profile is mostly specific for each bacterium, coherent with the dissimilar nature of P. aeruginosa and S. enterica (50). This poly(A)^+^ response is dramatically reduced in the F2, where differentially expressed genes are specifically involved in immune and defense response. Therefore, it is likely possible that in the second generation, specific transcriptional changes in poly(A)^+^ coding and noncoding genes are accumulated sufficiently to exceed the threshold, thus modulating developmental decisions to ensure survival. In this context, miRNAs and other sRNAs play key regulatory roles required for phenotypic and inter/transgenerational responses in C. elegans.

Dauer entry is a hard decision because it is metabolically and reproductively expensive for the animal. Therefore, we presumed that dauer-triggering signals should exceed a threshold that supports this choice. Time-dependent signaling molecules may be insufficient to reach a temporal threshold in the first generation of animals exposed to pathogens. For example, we know that dauer entry requires persistent intestinal colonization, which takes more than 48 h feeding on P. aeruginosa. Dauer entry is a complex process in which immune, metabolic, and stress signals are integrated at different levels of regulation.

## MATERIALS AND METHODS

### C. elegans and bacterial growth.

Wild-type, mutant, and transgenic C. elegans strains were grown at 20°C as previously described (51). All nematode strains were grown on Escherichia coli OP50-1 (resistant to streptomycin) before pathogen exposure. S. enterica serovar Typhimurium MST1 (ATCC 140828) and P. aeruginosa PAO1 (ATCC 15692) were used for infection protocols. All bacteria were grown overnight on Luria-Bertani (LB) plates at 37°C from glycerol stocks. The next morning, a large amount of the bacterial lawn is inoculated in LB broth and grown for 6 h at 250 rpm and at 37°C. Three milliliters of the resulting bacterial culture is seeded onto 90-mm NGM plates and allowed to dry for 36 h before worms are placed on them.

### C. elegans strains.

We used the following strains of the *Caenorhabditis* Genetics Center (CGC): wild type (N2), MT15454 [*mir-243* (*n4759*)], MT16060 [*mir-253* (*nDf64*)], DR27 [*daf-16* (*m27*)], VC3149 [*crh-2* (*gk3293*)], RB711 [*pqm-1* (*ok485*)], VC143 [*elt-3* (*gk121*)], and transgenic strains VT1474 [*Pmir-243*::*gfp* [*unc-119* (*ed3*) III; *maIs177*], OP201 [*unc-119* (*tm4063*) III; wgls201 (*pqm-1*::*TY1*::*EGFP*)], BC13136 [*crh-2* C27D5.4a*::gfp* (*dpy-5* (*e907*); sEx13136)], BC14266 [*dpy-5* (*e907*); sEx14266 (rCesF35E8.8::*GFP +* pCeH361)]. Regarding the pertinence of strains with mutations in TF gene, there are many available strains with mutations in *daf-16*. We chose DR27 because it was the strain with the strongest Daf-d phenotype under starvation. *pqm-1* (*ok485*) and *crh-2* (*gk3293*) are the only strains available in the CGC for those genes.

### Hypochlorite treatment.

To synchronize C. elegans and/or to obtain pure embryos, we prepared a 5% hypochlorite solution containing 20 ml of 1 M NaOH, 30 ml NaClO, and 50 ml H_2_O in a final volume of 100 ml. Plates with mostly gravid adults were washed with 1 ml of M9 (KH_2_PO_4_ [3 g], Na_2_HPO_4_ [6 g], NaCl [5 g], 1 M MgSO_4_, and H_2_O up to 1 liter) and transferred to an Eppendorf tube. The volume collected was centrifuged at 394 × *g*. One milliliter of the hypochlorite solution was added to the pellet. After 5 min of vigorous vortexing, the tube was centrifuged at 394 × *g* for 2 min. The pellet was washed with 1 ml of M9 solution and centrifuged at 394 × *g* and 2 min to discard the supernatant. The resulting pellet contains an embryo concentrate.

### Quantification of population and dauer larvae: dauer formation on pathogens.

The entire worm population on each plate was collected in 1 ml of M9. This initial stock was diluted 1:10 in M9. Ten microliters of this 1:10 dilution was used to count the total population of worms under a Nikon SMZ745 stereomicroscope. To quantify the number of dauers in each population, the initial stock was diluted 1:10 in a 1% sodium dodecyl sulfate (SDS) solution and maintained in constant agitation for 20 min (52). To count the number of total animals and dauers, 10 μl of this last dilution was placed on a glass slide and examined with a stereomicroscope. Each condition was scored three times (triplicates of one technical replicate), and dauers were plotted as a percentage of the total population of animals.

### C. elegans growth in pathogenic bacteria.

Five L4 (P0) wild-type worms or mutants (grown in E. coli OP50) were picked and transferred to a 90-mm-diameter plate seeded with 3 ml of P. aeruginosa PAO1, S. enterica serovar Typhimurium MST1, or E. coli OP50 control bacteria. In all cases, the bacterial lawn covered the plate. After 24 h, the F1 embryos were obtained by hypochlorite treatment. All obtained embryos were placed on a new plate with P. aeruginosa PAO1, S. enterica serovar Typhimurium MST1, or E. coli OP50. Animals were allowed to grow for 24 h until they reached the L2 stage. The total number of worms in the population and the percentage of dauer were quantified.

To obtain the F2, five L4 larvae were transferred from E. coli OP50 to a 90-mm-diameter plate with 3 ml of P. aeruginosa PAO1 or S. enterica serovar Typhimurium MST1. After 8 days, the total number of worms and dauer larvae were quantified. The number of bacteria seeded allowed animals to be well fed for the length of the experiment. In the case that worms starved, we discarded that experiment. Each assay was performed in three independent experiments (technical replicates) generating a biological replicate. A total of three biological replicates were considered for each analysis.

### Dual RNA-seq: sample preparation.

Wild-type C. elegans was cultured on 60-mm-diameter petri dishes with NGM medium seeded with 500 μl of E. coli OP50 and maintained at 20°C. After 3 days, mixed stage animals were treated with bleaching solution and embryos were deposited in new dishes. Forty-eight hours later, most individuals were in the L4 stage. Five L4 worms were transferred to 90-mm plates previously seeded with 3 ml of E. coli OP50, S. enterica serovar Typhimurium MST1, or P. aeruginosa PAO1. In all cases, the bacterial lawn covered the plate. Worms were allowed to grow at 20°C for 24 h. After that time, all animals on the plate were subjected to hypochlorite treatment. F1 embryos were collected in 1 ml of M9 and centrifuged at 394 × *g*. The embryos obtained were placed on a new 90-mm plate with 3 ml of bacteria. After 24 h, animals were collected with M9 for total RNA extraction. Worms on the other three plates were allowed to grow for another 48 h until the F1 was gravid. F2 progenies were collected in the same way as the F1 and placed on a separate plate with the same species of bacteria. Animals were collected for RNA extraction 24 h later. Each condition was performed in triplicates obtaining a total of 18 samples (F1 and F2 in E. coli OP50, P. aeruginosa PAO1, or S. enterica serovar Typhimurium MST1).

### RNA extraction.

**(i) RNA extractions of colonizing bacteria and C. elegans.** Worms were washed off the plates with 1 ml of M9, centrifuged at 394 × *g* for 2 min, and resuspended at least five times with M9. RNA purification was performed using TRIzol (Life Technologies) following the manufacturer’s instructions. For RNA extraction, samples were mechanically lysed by vortexing with 4-mm steel beads for no more than 5 min. Each condition was performed in triplicates. Several biological replicates were mixed to reach the required concentration of 1 μg/μl of total RNA.

**(ii) mRNA library preparation and sequencing.** Total RNA was isolated from synchronized F1 and F2 C. elegans populations feeding on nonpathogenic E. coli OP50 and pathogens P. aeruginosa PAO1 and S. enterica serovar Typhimurium MST1 as explained above. After digestion with DNase I (Invitrogen), RNA concentration was measured using Quant-iT RiboGreen RNA assay kit (Life Technologies). The integrity of RNA was determined on the Agilent 2100 bioanalyzer (Agilent Technologies). mRNA libraries were prepared with the IlluminaTruSeq RNA sample preparation kit (Illumina) according to the manufacturer's protocol. The quality and size distribution of the libraries were evaluated with the Agilent 2100 bioanalyzer using a DNA 1000 chip (Agilent Technologies) and quantified using the KAPA Library Quantification kit for Illumina Platforms (Kapa Biosystems) on the Step One Plus real-time PCR system (Applied Biosystems).

The C. elegans mRNA libraries were sequenced using the HiSeq Illumina platform (BGI) with paired-end sequencing (2 × 100 bp, BGI). C. elegans and bacterial small RNA were sequenced using the Mi-Seq Illumina platform at the Center for Genomics and Bioinformatics (CGB), Universidad Mayor.

**(iii) sRNA library construction and sequencing.** Samples were prepared and sequenced at CGB. Libraries were constructed with the TruSeq sRNA Sample Preparation kit (Illumina), according to the manufacturer´s instructions. For quality control, cDNA libraries were run on a high-sensitivity DNA chip using the Agilent 2100 bioanalyzer (Agilent Technologies) according to the manufacturer’s instructions. An agarose gel library size selection was performed to recover RNAs shorter than 200 nucleotides. The library was quantified using a high-sensitivity DNA chip. The sRNA libraries were sequenced in an Illumina MiSeq sequencer using the MiSeq reagent v2 50-cycle kit with single-end sequencing (1 × 36 bp). For each biological condition, three libraries were constructed from biological replicates. The raw data generated have been deposited with links to BioProject accession number PRJNA659467 in the NCBI BioProject database (https://www.ncbi.nlm.nih.gov/bioproject/).

### Bioinformatic analysis.

**(i) mRNA transcriptomics of C. elegans.** *(a) Data preprocessing and quality control.* Trimming was performed with Trimmomatic v. 0.36 (53). Reads with a quality score (Phred score) of less than 35 and a read length of less than 36 were removed.

*(b) Mapping and read count.* Reads were aligned using Tophat (54) with default parameters. Reads were quantified using HTSeq count (55).

*(c) Differential expression.* Differential expression was determined using EdgeR (56) and DeSeq (57). Differentially expressed genes were defined as those with adjusted *P* value (padj) of <0.05 by either method.

*(d) Enrichment analysis: Gene Ontology analysis*. Gene Ontology (GO) analysis was performed using the enrichment tool in wormbase (58).

**(ii) Small RNA transcriptomics of C. elegans.** *(a) Data preprocessing and quality control.* Quality visualization was made with FastQC (http://www.bioinformatics.babraham.ac.uk/projects/fastqc). Illumina small 3′ adaptors were removed with Cutadapt version 1.13 (59). Reads with an average quality over four bases lower than 30, as well as reads shorter than 17 bp were discarded with trimmomatics (53).

*(b) Mapping*. For each sample, the reads were aligned against the C. elegans genome PRJNA13758 WS267 using bowtie2 version 2.2.6 (60). We chose a 17-bp seed length, which was the length of the shorter read. The seed interval was calculated as $f(x)=0+2.5\sqrt{x}$ where *x* is the read length (roughly two seeds per read as reads are 36 or smaller). For greater sensitivity, we set the number of mismatches allowed in a seed to 1. By default, 15% of ambiguous characters per read is allowed. A bam file was produced for each sample.

*(c) Defining features and counting reads.* We developed a strategy to detect both known and unannotated transcripts from bacteria and C. elegans. To do that, we chose to make a reference annotation based on observed expression peaks. Some reads overlap annotated genes, others show more than one peak in annotated regions, and many peaks are located in unannotated regions. We selected those genomic areas covered by mapped reads to define customized features, named transcriptional peaks (TPs) defined as a genomic area that shows a peak of expression (more than 10 per base) and that may correspond to direct transcription or fragments of a longer transcript. This was done by merging bam files from each sample using SAMTools version 0.1.19 (61). Afterward we kept only features between 17 and 150 nucleotides with an average coverage of 10 or more reads by nucleotide. The features obtained for both strands were gathered and sorted to create a custom GFF file for further analysis. Next, we counted the reads against those custom GFF files. For comparison with databases, we intersected TPs with reported annotations and classified them according to their genomic context.

*(d) Comparison with annotated genes*. To see how TPs matched with annotated genes, we compared them to the Ensembl database, by intersecting the GTF file of C. elegans (PRJNA13758, WS267) with our custom GFF file, using BEDTools (62). On the basis of this result, we classified TPs as novel (in intergenic unannotated regions), nested or overlapping annotated features, and sense or antisense to a known feature.

*(e) Differential expression analysis between conditions*. For each sample, read count was performed with featureCounts (63) from the Bioconductor Rsubread package with default parameters. Then, the count matrix was used to perform differential expression analysis in R (version 3.3.2) between worms fed with three different bacteria of each generation using DeSeq2 (64) version 1.4.5. The samples of animals feeding on pathogenic strains P. aeruginosa PAO1 and S. enterica serovar Typhimurium were compared with E. coli OP50. For each condition, the first and second generations were compared.

We conducted the previous analysis using a custom-made bash and R scripts, available at https://github.com/mlegue/gabaldon2020.

*(f) Prediction of mir-243-3p targets*. We used the IntaRNA tool version 2.0 (22); http://rna.informatik.uni-freiburg.de/IntaRNA/Input.jsp. We adjusted parameters to force the longest possible seed with perfect complementarity. We started using the maximal admitted seed length (SeedBP = 20) and run the analysis with all seed length above default (SeedBP = 8) with no restrictions on seed energy. We also set the temperature to 20°C at which experiments were performed. We performed the interactions between *mir-243*-*3p* and the downregulated mRNA genes [poly(A) genes] in our data set. We also evaluate with the same parameters the interaction with Y47H10A.5, a validated *mir-243-3p* target.

### Quantification of differential expression by RT-PCR.

*(a) Total RNA extraction.* Five L4 (P0) worms were placed in 90-mm NGM plates seeded with P. aeruginosa PAO1. After 24 h, the total population was collected with 1 ml of M9 and centrifuged at 394 × *g* for 2 min. The pellet was treated with hypochlorite solution (see above). All resulting F1 embryos were placed on a new plate with P. aeruginosa PAO1 for 24 h and collected as L2. Collection is done with M9 in an Eppendorf tube. Contents were centrifuged at 394 × *g* for 2 min. L2 pellet was washed five times with M9 to eliminate the most bacteria from the sample. The pellet of synchronized worms (L2) was used for RNA extraction.

F2 animals were obtained from the same L4 P0 fed with P. aeruginosa and used to generate the F1 as explained above. F1 embryos were placed in a new 90-mm plate with 3 ml of P. aeruginosa PAO1. After 72 h, the total population was collected with 1 ml of M9 and centrifuged at 394 × *g* for 2 min. The pellet was treated with hypochlorite solution. F2 embryos were placed on a new plate with P. aeruginosa for 24 h. The L2 worms were washed off the plate with M9 and centrifuged at 394 × *g* for 2 min. Five washes with M9 were necessary to eliminate most bacteria from the sample. The pellet of synchronized worms (L2) was treated with 4-mm steel beads and 1 ml of RNA-Solv reagent (Omega Bio-Tek). The mix was vortexed for 5 min. RNA extraction was performed according to the manufacturer’s specifications. Total RNA concentration was quantified with Tecan’s NanoQuant Plate. Each condition was performed in biological triplicates.

*(b) cDNA synthesis.* Using the extracted total RNAs, cDNA synthesis was performed with the MIR-X miRNA First Strand Synthesis from TaKaRa following the manufacturer’s specifications.

*(c) Real-time PCR (qPCR).* The cDNA samples (concentration 200 ng/μl) were used as a template to perform quantitative PCR (qPCR) with the primers Qiagen Ce_miR-243_1 miScript primer assay (MS00019481). The qPCR was performed with the MIR-X miRNA qRT-PCR TB Green kit from TaKaRa. To calculate the relative fold of expression of *mir-243-3p* between generations and genotypes, we used ΔΔ*C_T_* calculations according to the reference unit mass method (65). This method was based on the comparative use of the test sample against a calibrator (U6). Values less than 1 indicated the negative expression relation with respect to the control, and a ratio greater than 1 indicated the times above the sample with respect to the control.

### Quantification of *gfp* expression.

Expression levels of *pqm-1* and *crh-2* genes and the promoter of *mir-243* were quantified by using wild-type animals expressing *gfp*. *gfp* was quantified in F1 and F2 L2 fed with P. aeruginosa PAO1 and E. coli OP50. Worms were taken individually with a mouth pipette and placed in a bed of agarose with levamisole (20 mM) to immobilize them. To quantify *gfp* expression, we photographed entire animals with a Nikon Eclipse Ni microscope and a 40× objective at 1/320 s (*mir-243::gfp*) and 1/10 s (*pqm-1* and *crh-2*) exposure time, ISO 200/3200 speed (white light and laser, respectively) and focal length of 50 mm. For all markers, photos of entire animals were taken. Green fluorescent protein (GFP) quantification was done considering the signal from the entire animal. Image analysis was performed using ImageJ. Prior to the analysis, images are converted to 8-bit format and a threshold between 1 and 1.2.

### Statistical analysis.

Statistical analyses were conducted using one- or two-way analysis of variance (ANOVA) with *post hoc* tests. For the differential expression analysis, statistical significance was considered with a *P* value of <0.05. All experiments were repeated at least three times using technical replicates in each experiment.

### Data availability.

Raw data were deposited in the NCBI under BioProject no. PRJNA659467.
