# Supplementary material for: Intergenerational Pathogen-Induced Diapause in Caenorhabditis elegans Is Modulated by mir-243
Source: mBio. 2020 Sep 22;11(5):e01950-20. doi: 10.1128/mBio.01950-20 (PMC7512553; doi:10.1128/mBio.01950-20)
Supplement: FIG S1 [file mBio.01950-20-sf001.pdf]

Figure S1

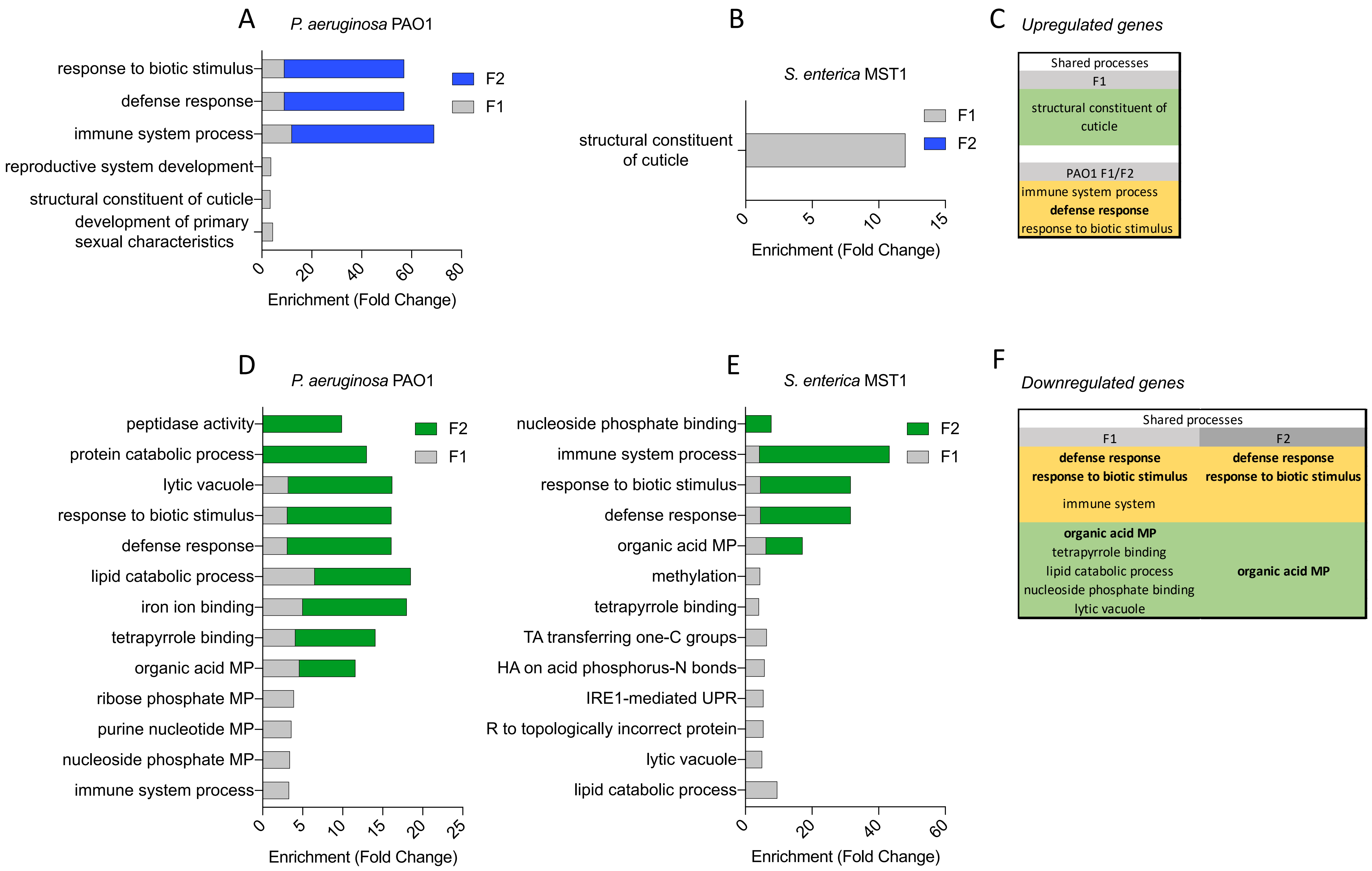

Fig. S1. Enrichment by GO term of upregulated (A,B) and downregulated (D,E) in animals feeding on *P. aeruginosa* PAO1 and *S. enterica* serovar Typhimurium MST1 in two generations. C, F Summary of shared GO terms in F1 and F2 in up (C) and downregulated (F) genes. MP, metabolic process; TA, transferase activity; HA, hydrolase activity; N, Nitrogen; C, Carbon; R, response; P, phosphate; UPR, Unfolded Protein Response.
